# Intranasal Delivery of Apelin-13 Is Neuroprotective and Promotes Angiogenesis After Ischemic Stroke in Mice

**DOI:** 10.1177/1759091415605114

**Published:** 2015-09-10

**Authors:** Dongdong Chen, Jinhwan Lee, Xiaohuan Gu, Ling Wei, Shan Ping Yu

**Affiliations:** 1 Deptartment of Anesthesiology, Emory University School of Medicine, Atlanta, GA, USA; 2 Center for Visual and Neurocognitive Rehabilitation, Atlanta Veterans Affair Medical Center, Decatur, GA, USA; 3 Department of Neurology, Emory University School of Medicine, Atlanta, GA, USA

**Keywords:** apelin-13, neuroprotection, angiogenesis, inflammation, functional recovery, ischemic stroke

## Abstract

Apelin is a peptide originally isolated from bovine stomach tissue extracts and identified as an endogenous ligand of the APJ receptor; recent work showed that apelin ameliorates the ischemic injury in the heart and the brain. Being an analogue to the angiotensin II receptor, the apelin/APJ signaling may mediate angiogenesis process. We explored the noninvasive intranasal brain delivery method and investigated therapeutic effects of apelin-13 in a focal ischemic stroke model of mice. Intranasal administration of apelin-13 (4 mg/kg) was given 30 min after the onset of stroke and repeated once daily. Three days after stroke, mice received apelin-13 had significantly reduced infarct volume and less neuronal death in the penumbra. Western blot analyses showed upregulated levels of apelin, apelin receptor APLNR, and Bcl-2 and decreased caspase-3 activation in the apelin-13-treated brain. The proinflammatory cytokines tumor necrosis factor-alpha, interleukin-1β, and chemokine monocyte chemoattractant protein-1 mRNA increased in the ischemic brain, which were significantly attenuated by apelin-13. Apelin-13 remarkably reduced microglia recruitment and activation in the penumbra according to morphological features of Iba-1-positive cells 3 days after ischemia. Apelin-13 significantly increased the expression of angiogenic factor vascular endothelial growth factor and matrix metalloproteinase-9 14 days after stroke. Angiogenesis illustrated by collagen IV + /5-bromo-2′-deoxyuridin + colabeled cells was significantly increased by the apelin-13 treatment 21 days after stroke. Finally, apelin-13 promoted the local cerebral blood flow restoration and long-term functional recovery. This study demonstrates a noninvasive intranasal delivery of apelin-13 after stroke, suggesting that the reduced inflammatory activities, decreased cell death, and increased angiogenesis contribute to the therapeutic benefits of apelin-13.

## Introduction

Stroke is a leading cause of human death and disability in the adult population in the United States and around the world. Among all stroke patients, 87% suffer from ischemic stroke (Roger et al., 2012). So far effective stroke treatments are still limited to thrombolytic therapy using tissue plasminogen activator with a narrow time window of 4.5 hr after the onset of an ischemic attack (Shobha et al., 2011; Jauch et al., 2013). Thus, stroke represents a clinical entity that requires more innovative treatments both for acute neuroprotection and for regenerative tissue repair.

Apelin was originally isolated from bovine stomach tissue extracts. It has been identified as an endogenous ligand of the APJ receptor, a G protein-coupled receptor related to angiotensin receptor AT1 (Lee et al., 2000a). Apelin is derived from a 77-amino acid length precursor peptide that can be cleaved by angiotensin-converting enzyme 2 into active apelins, including apelin-36 (42–77), apelin-17 (61–77), and apelin-13 (65–77; Lee et al., 2000b). Apelin-13 has completely conserved 13 C-terminal amino acids that are cross all species and exhibits the highest biological potency, including cardiomyocytes protection (Hosoya et al., 2000; Kleinz and Davenport, 2005; Simpkin et al., 2007). The active apelins are widely distributed in various organs and tissues, including the brain, lungs, testis, and uterus, and are highly expressed in the cardiovascular system. In the brain, apelins are widely expressed in neuronal cell bodies and fibers throughout the entire neuroaxis (Cheng et al., 2012). In neurological diseases, apelin level is significantly altered in the central nervous system. For example, apelin is significantly elevated in the epileptogenic temporal neocortex and absent in glial cells of temporal lobe epilepsy patients (Zhang et al., 2011).

Apelin receptor AGTRL1 was shown to associate with the development of ischemic stroke in the most recent genome-wide association study for ischemic stroke (Hata et al., 2011). As a neuropeptide, apelin exhibits neuroprotective function in both *in vitro* and *in vivo* studies. Pretreatment with apelin-13 or apelin-36 peptides, alone or in combination, increased hippocampal neuronal survival from 25% to 50% to 75% after HIV-induced excitotoxic injury (O’Donnell et al., 2007). Our previous *in vitro* study also showed that apelin-13 reduced serum deprivation-induced reactive oxygen species generation, mitochondria depolarization, cytochrome c release, and activation of caspase-3. We showed that apelin-13 could regulate cell survival kinases the protein kinase B (PKB, also known as AKT) and extracellular signal-regulated kinase (ERK)1/2 in cultured cortical neurons (Zeng et al., 2010). Most recently, apelin-13 was also demonstrated to protect brain from ischemia/reperfusion (IR) injury through activation of AKT and ERK1/2 signaling pathways in a mouse focal transient cerebral ischemia model (Yang et al., 2014). In a cerebral middle artery occlusion filament stroke model, apelin-36 reduced cell death and cerebral edema (Khaksari et al., 2012; Gu et al., 2013).

APJ has high-sequence homology with the angiotensin II type I receptor, but it binds to apelin instead of angiotensin II (O’Dowd et al., 1993; Lee et al., 2000a). Due to its similarity to the angiotensin II receptor, the functions of APJ have been widely studied on the cardiovascular system. Increasing evidence shows that the apelin/APJ signaling mediates the angiogenesis process. Overexpression of apelin increased Sirt3, vascular endothelial growth factor (VEGF)/VEGFR2, and angiopoietin-1 (Ang-1)/Tie-2 expression and the density of capillary and arteriole in the heart of diabetic mice (Zeng et al., 2014). Inhibition of apelin expression switched endothelial cells from proliferative to mature state in pathological retinal angiogenesis (Kasai et al., 2013). The proangiogenic role of apelin was also demonstrated in myocardial IR injury and murine hindlimb ischemia model. The loss of apelin impaired the angiogenesis and functional recovery, and exacerbated myocardial IR injury, while the elevation of apelin expression induced by adeno-associated virus transduction benefited the postischemic hindlimb perfusion (Qin et al., 2013; Wang et al., 2013). All the above evidence indicates the potential regenerative effects of apelin and a therapeutic application after ischemia. However, in all these *in vivo* studies, apelin was administered through lateral cerebral ventricle injection, which is highly invasive and less feasible in clinical conditions. As a potential protective drug for ischemic stroke treatment, it is important to seek for a noninvasive method to deliver apelin.

Intranasal administration is a noninvasive method to direct protein and peptide drugs into the brain by utilizing the olfactory neuronal distribution pathways in the cribriform plate, which leads to direct nose-to-brain drug distribution, bypasses the blood–brain barrier (BBB), and directly guides therapeutics to the brain (Hanson and Frey, 2008; Dhuria et al., 2010). Intranasal administration can directly transfer protein and peptides to the brain in similar or higher concentrations than that can be obtained by systemic administration (Scafidi et al., 2014). In this investigation, we tested the hypothesis that the neuroprotective effects of apelin-13 can be achieved by noninvasive intranasal delivery via reducing the infarct formation and inflammatory activities after ischemic stroke, leading to a long-term angiogenesis and functional recovery after stroke.

## Materials and Methods

### Focal Ischemic Stroke Model

All animal experiments and surgery procedures were approved by the Institutional Animal Care and Use Committee and met NIH standard. Focal cerebral ischemia was induced in adult male C57/BL6 mice, which were purchased from Charles River Laboratories and housed at Emory University in standard cages in 12 hr light/12 hr dark cycles. Surgery procedures were modified from a previously described rat protocol (Wei et al., 2005). Briefly, animals were subjected to 4% chloral hydrate anesthesia, and the distal branches of the right middle cerebral artery (MCA) were permanently ligated by a 10-0 suture (Surgical Specialties CO., Reading, PA, USA). The creation of the sensorimotor cortical ischemia was completed by bilateral occlusion of the common carotid arteries (CCA) for 7 min followed by reperfusion. During surgery and recovery periods, body temperature was monitored and maintained at 37.0 ± 0.5℃ using a temperature control unit and heating pads.

### Intranasal Drug Administration

Intranasal drug delivery was performed as previously described with minor modifications (Scafidi et al., 2014). All animals (25–28 g) received 100 U hyaluronidase (Sigma, St. Lous, MO) dissolved in sterile phosphate-buffered saline 30 min prior to the apelin-13 administration. By catalyzing the hydrolysis of hyaluronan, hyaluronidase can increase tissue permeability. Apelin-13 (Sigma-Aldrich, St Louis, MO, USA) was dissolved in sterile saline at 1 mg/ml concentration; 5–10 µl drops containing the saline or apelin-13 (1 mg/ml) were carefully placed on one nostril of conscious animal, allowing it to be snorted, alternating the nostrils with 1 min intervals. A total volume of 100 µl saline or apelin-13 (1 mg/ml, equivalent to 4 mg/kg) was administered to each ischemic animal 30 min after stroke onset and followed by once daily until animals were sacrificed. 5-Bromo-2′-deoxyuridin (BrdU, 50 mg/kg; Sigma) was administered intraperitoneally to label proliferating cells since Day 3 after stroke until animals were sacrificed.

### Quantification of Infarct Volume

Infarct volume was assessed 3 days after ischemia by 2,3,5-triphenyltetrazolium chloride (TTC) staining. The brains were removed and placed in a coronal brain matrix and then sliced into 1-mm sections. Slices were incubated in 2% TTC (Sigma) solution at 37℃ for 5 min and then stored in 10% buffered formalin for 24 hr. The digital images of the caudal aspect of each slice were obtained using a flatbed scanner. Infarct, ipsilateral hemisphere, and contralateral hemisphere areas were measured using Image J software (NIH, Bethesda, MD, USA). Infarct volume was calculated using the indirect method (Swanson et al., 1990).

### Terminal Deoxynucleotidyl Transferase Biotin-dUPT Nick-End Labeling

A terminal deoxynucleotidyl transferase biotin-dUPT nick-end labeling (TUNEL) assay kit (DeadEnd Fluorometric TUNEL system; Promega, Madison, WI, USA) was used to assess cell death by detecting fragmented DNA in 10 -µm-thick coronal fresh frozen sections. Briefly, after fixation in 10% buffered formalin for 10 min and then ethanol:acetic acid (2:1) solution for 5 min, permeabilization was done in 0.2% Triton-X 100 solution. Brain sections were incubated in equilibration buffer for 10 min and then in recombinant terminal deoxynucleotidyl transferase (rTdT) and nucleotide mixture at 37℃ for 60 min in the dark. Reactions were terminated by 2 × SSC solution for 15 min. Nuclei were counterstained with Hoechst 33342 (1:20,000; Molecular Probes, Eugene, OR, USA) for 5 min.

### Immunofluorescence Staining

Fresh frozen brain sections were stained by using standard protocols for NeuN (Millipore, Billerica, MA, USA) to label neurons, collagen IV (Millipore) to label vessels, BrdU (AbD Serotec, Oxford, UK) to label newborn cells, and occludin (Life technologies, Grand Island, NY, USA) to detect the BBB. Pictures were taken by using fluorescence microscopy along the length of the penumbra region defined morphologically as the region just outside the stroke core. Perfusion-fixed brain sections were used for Iba-1 (Biocare Medical, Concord, CA, USA) and F4/80 (AbD Serotec) staining, followed by standard staining protocol. Staining was visualized by fluorescent microscopy (BX61; Olympus, Tokyo, Japan). For systematic random sampling in design-based stereological cell counting, six coronal brain sections per mouse were selected, spaced 90 µm apart across the same region of interest in each animal. For multistage random sampling, six fields per brain section were randomly chosen in the peri-infarct/penumbra region of the brain. All counting assays were performed under blind condition. The area of positive staining was measured using the fraction area measurement function of NIH Image J software.

The data analysis of reactive microglia in the penumbra region was based on the morphological assessment of Iba-1-positive cells according to published method (Soltys et al., 2001). Briefly, based on the length of branches, thickness of branches, and cell body volume, the Iba-1-positive cells were categorized to three classes: (a) ramified microglia (surveillant/resting microglia), characterized by small round or oval cell bodies containing a small volume of cytoplasm; (b) hypertrophied microglia, which had larger cell bodies and thicker processes than ramified microglia; and (c) bushy microglia, which had numerous but short processes forming thick bundles around their swollen cell bodies. Hypertrophied and bushy Iba-1-positive cells were identified as activated microglia.

### Western Blot Analysis

The peri-infarct/penumbra region was defined as previously described by a 500-µm boundary extending from the edge of the infarct core, medial, and lateral to the infarct (Ohab et al., 2006). Tissue samples were taken from the peri-infarct/penumbra region of the cortex, and proteins were extracted by homogenization in protein lysis buffer (25 mM Tris-HCl [pH 7.4], 150 mM NaCl, 5 mM EDTA, 0.1% SDS, 2 mM sodium orthovanadate, 100 mM NaF, 1% Triton, leupeptin, aprotinin, and pepstatin). Protein (30 µg) from each sample was loaded into a gradient gel and run at constant current until protein markers had adequately separated. They were transferred onto polyvinyl difluoride membranes that were then probed by using standard protocols (Choi et al., 2012). Primary antibodies Bcl-2 (1:1000; Cell signaling, Boston, MA, USA); cleaved caspase-3 (1:500; Cell signaling); VEGF (1:100; Santa Cruz, Dallas, Texas, USA); brain-derived neurotrophic factor (BDNF; 1:500; Santa Cruz); matrix metalloproteinase-9 (MMP9; 1:500; Santa Cruz); Apelin (C-13; 1:100; Santa Cruz); APJR-1 (H-300; 1:100; Santa Cruz); and mouse β-actin antibody (1:6000; Sigma) were applied overnight at 4℃. Alkaline phosphatase-conjugated secondary antibodies were applied for 1 to 2 hr at room temperature. Alkaline phosphatase-conjugated antibodies were developed by using nitro-blue tetrazolium and 5-bromo-4-chloro-3’-indolyphosphate solution. The intensity of each band was measured and subtracted by the background using NIH Image J software. The expression ratio of each target protein was then normalized against β-actin.

### Gelatin Zymography Assay

MMP assay kit (Biomedical Research Service Center, Buffalo, NY, USA) was used to detect the activity of MMP9. The procedure for gelatin zymography assay followed the instructions provided in the kit. Briefly, the tissue was homogenized, and protein was extracted in cell lysis solution. Protein sample (50 µg) was loaded and run in 10% SDS-polyacrylamide gel copolymerized with substrate (1 mg/ml of gelatin) until protein markers had adequately separated. After washing with 1 × MMP wash buffer for 15 min, the gel was incubated in 1 × MMP reaction buffer at 37℃ for 48 hr. The gel was then stained in a 3% Coomassie Brilliant Blue G250 solution for 90 min and destained in 10% methanol/10% acetic acid solution until the MMP bands fully develop against the blue background. MMP9 band intensity was quantified using the NIH Image J software.

### Isolation of Total RNA and RT-PCR

Total RNA from tissues of experimental brains was isolated according to manufacturer’s instruction (Life Technologies). RNA integrity was confirmed by detection of 28 s and 18 s rRNA band. RNA was confirmed to be free of genomic DNA contamination by PCR in the absence of reverse transcriptase. The RNA samples (1 µg) were reverse transcribed in 20 µl of a reaction mixture containing 2 × RT buffer and 20 × RT enzyme mix according to manufacturer’s instruction (Life Technologies) at 37℃ for 60 min. The samples were then incubated at 95℃ for 5 min and transferred to 4℃. RT product (1 µl) was subjected to PCR amplification with 10 pmole primer, 10 × standard Taq reaction buffer, 10 mM dNTP, and 0.625 unit Taq polymerase in 20 µl PCR reaction buffer (New England Biolabs Inc., Ipswich, MA, USA). PCR primers were used as follows (5′–3′): tumor necrosis factor-alpha (TNF-α, GATCTCAAAGACAACCAACTAGTG (forward) and CTCCAGCTGGAAGACTCCTCCCAG (reverse); interleukin (IL)-1β, TCGGCCAAGACAGGTCGCTCA (forward) and TGGTTGCCCATCAGAGGCAAGG (reverse); IL-6, GAGGATACCCCCAACAGACC (forward) and AAGTGCATCATCGTTGTTCATACA (reverse); IL-10, CACCCACTTCCCAGTCGGCCA (forward) and TGCTTCTCTGCCGGCATCA (reverse); macrophage inflammatory protein (MIP)-1α, ATGAAGGTCTCCACCACTG (forward) and GCATTCAGTTCCAGGTCA (reverse); monocyte chemoattractant protein-1 (MCP-1), ATGCAGGTCCCTGTCATG (forward) and GCTTGAGGTGGTTGTGGA (reverse); 18 s, GACTCAACACGGGAAACCTC (forward) and ATGCCAGAGTCTCGTTCGTT (reverse). PCR mixtures were heated to 95℃ for 10 min and cycled 30 to 37 times for each primer; cycles consisted of 95℃ for 15 s, 60℃ for 1 min, and 72℃ for 30 s. After additional incubation at 72℃ for 10 min, the PCR samples were transferred to 4℃. PCR products were subjected to electrophoresis in 2% agarose gel with ethidium bromide. Relative intensity of a PCR band was analyzed using InGenius3 manual gel documentation systems (Syngene, Frederick, MD, USA).

### Local Cerebral Blood Flow Measurement

Laser scanning imaging was used to measure local cerebral blood flow (LCBF) as previously described (Li et al., 2007) at three time points: immediately before MCA ligation, right after occlusion, during the 7 min bilateral CCA ligation and 21 days after ischemia. Briefly, animals were anesthetized with an injection of 4% chloral hydrate solution, and an incision was made to expose the skull above the territory of the right MCA. The laser was centered over the right coronal suture. Different from the conventional Laser Doppler probe that measures a small point of blood flow, the scanner method measures a 2.4 × 2.4 mm square area using the Laser Doppler perfusion imaging system (PeriFlux System 5000-PF5010 LDPM unit, Perimed, Stockholm, Sweden). This scanning measurement largely avoids inaccurate or bias results caused by inconsistent location of the laser prob. Data were analyzed using the LDPI Win 2 software (Perimed AB, Stockholm, Sweden).

### HomeCageScan and TopScan Behavioral Assessment

The behavioral changes of experimental mice were monitored and analyzed using the HomeCageScan system (Clever Sys Inc., Reston, VA, USA) for 12 hr starting from 2 days to 3 days after stroke. The system had four cameras that monitored four cages, with each cage (191 mm × 292 mm × 127 mm) containing one mouse. The behavior patterns were continuously recorded for 12 hr at dark cycle). After finishing the recording, the videos were analyzed by the HomeCage Software 3.0 (Clever Sys Inc.). Three days after stroke, the motor function of the mouse was measured by the TopScan system (Clever Sys Inc.). All procedures were carried out in a square open-field test chamber (50 cm × 50 cm × 50 cm). Mice were placed in the center of the chamber and allowed to move around freely for 1 hr. Behaviors such as distance, velocity, and the entries into the center area were captured and analyzed using the TopScan software 2.0 (Clever Sys Inc.). The arena was cleaned with 70% ethanol after each mouse completed a session.

### Adhesive Removal Test

The adhesive removal test measures sensorimotor function as previously described (Bouet et al., 2009). A small adhesive dot was placed on each forepaw, and the time (seconds) needed to contact and remove the sticker from each forepaw was recorded. Mice were trained three times before stroke surgery to ensure that they have normal sensorimotor function. Recording was stopped if the animal failed to contact the sticker within 2 min. The test was performed three times per mouse, and the average time was used in the analysis at 21 days after stroke.

### Statistical Analysis

Student’s *t* test was used for the comparison of two experimental groups. Multiple comparisons were done using one-way analysis of variance (ANOVA) followed with Tukey test for multiple pairwise examinations. Two-way ANOVA followed with Bonferroni posttests was used for comparisons of multiple groups with multiple time points. Changes were identified as significant if *p* was less than .05. Mean values were reported together with the standard error of the mean.

## Results

### Apelin-13 Reduced the Infarct Volume After Ischemic Stroke

**Figure 1.**
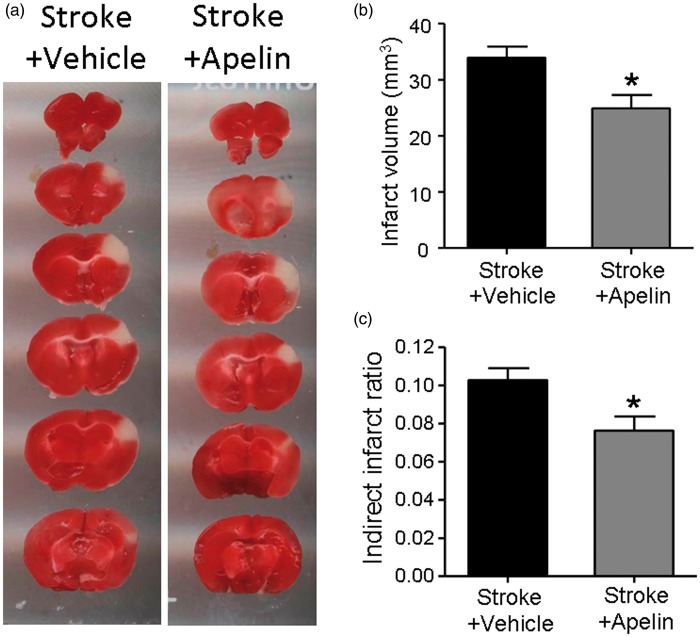
Apelin-13 reduced the infarct volume after ischemic stroke. Apelin-13 was administered 30 min after barrel cortex focal ischemic stroke onset and followed by once daily for 3 days after stroke. TTC staining was performed at 3 days after stroke to evaluate the infarct formation after stroke (a). Apelin-13 treatment significantly reduced the indirect infarct volume (b) and indirect infarct ratio (c). Data were represented as mean ± *SEM*, *p < .05; *n* = 12 in stroke + vehicle group and *n* = 14 in stroke + apelin group. TTC = 2,3,5-triphenyltetrazolium chloride.

Adult male mice were subjected to focal cerebral ischemia targeting the right sensorimotor cortex (Wei et al., 2005). Thirty minutes after the onset of ischemia, randomly assigned animals received saline vehicle control or apelin-13 (4 mg/kg) treatment via the intranasal route. The treatments were repeated once daily until the day of sacrifice. Three days after the ischemic stroke, brain coronal sections were analyzed for infarct formation using TTC staining. In stroke control mice, the cerebral ischemia induced a significant infarction in the right sensorimotor cortex (10.27 ± 0.63% infarction of the ipsilateral hemisphere). Apelin-13 treatment significantly reduced the infarct volume by 25.8% compared with the stroke control group (Figure 1(a) to (c)).

**Figure 2.**
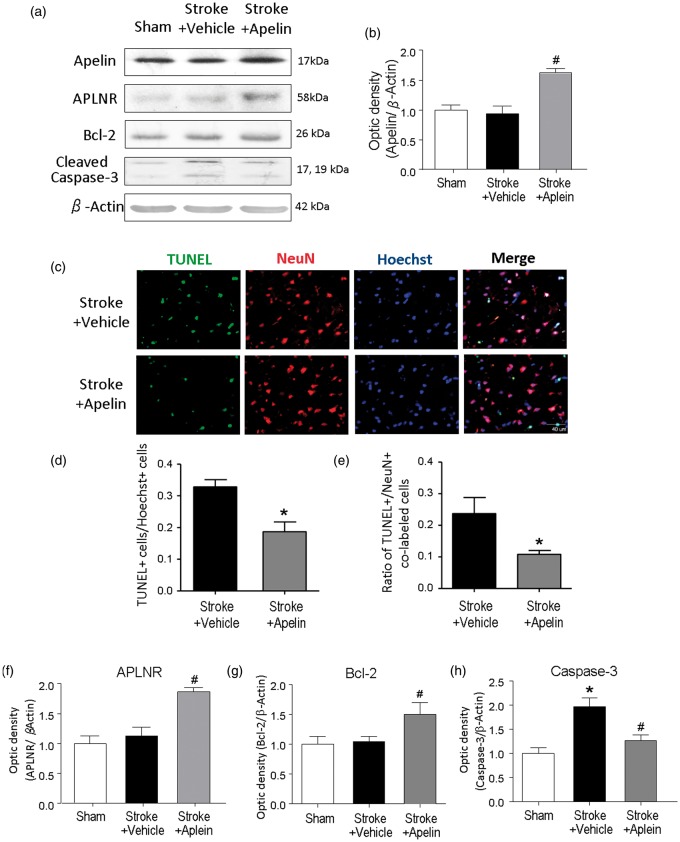
Apelin-13 reduced neuronal cell death in the ischemic brain. (a) Western blot assay was performed to detect the protein level of apelin in the ipsilateral cortex and the protein level of APLNR, Bcl-2, and cleaved caspase-3 in the penumbra region at 3 days after stroke. (b) Quantified data showed elevated level of apelin in stroke animals 30 min after intranasal delivery of apelin-13. #*p* < .05 versus stroke + vehicle; *n* = 3 in each group. (c) TUNEL (green) and neuronal marker NeuN (red) were stained to examine the neuronal cell death at 3 days after stroke. The TUNEL+/NeuN+ colabeled cells indicate the dead neurons. (d and e) The total number of TUNEL-positive cells was counted in the penumbra region. The ratio of TUNEL-positive cells to Hoechst-positive (blue) cells was then calculated. The number of TUNEL+/NeuN+ colabeled cells was also counted and the ratio of TUNEL+/NeuN+ colabeled cells was calculated. Apelin-13 remarkably reduced the ratio of TUNEL-positive cells and the ratio of TUNEL and NeuN colabeled cells in the penumbra region 3 days after stroke. *p < .05 versus stroke + vehicle; *n* = 5 each group. (f to h) Quantified Western blot data showing the protein expression levels of APLNR, Bcl-2, and cleaved caspase-3 in the penumbra region 3 days after stroke. The level of cleaved caspase-3 expression increased in stroke control animals. Stroke animals that received apelin-13 treatment showed significantly higher levels of APLNR, Bcl-2, and lower level of cleaved caspase-3 than those in stroke control animals (f to h). *p < .05 versus sham, #*p* < .05 versus stroke + vehicle; *n* = 3 in sham group, *n* = 3–4 in stroke + vehicle group, *n* = 3–4 in stroke + apelin group. TUNEL = terminal deoxynucleotidyl transferase biotin-dUPT nick-end labeling.

To verify that intranasally administered apelin-13 reached the brain, the apelin level in the brain was measured using Western blot assay. As early as 30 min after intranasal administration, the apelin level was noticeably higher in the ipsilateral cortex compared with that in stroke control animals (Figure 2(a) and (b)).

### Apelin-13 Protects Brain Against Apoptotic Cell Death After Ischemic Stroke

To understand the mechanism of protective effect of apelin-13 at the cellular level, we performed the TUNEL staining to detect the apoptotic cell death at 3 days after stroke. In apelin-13 treatment group, there were significantly fewer TUNEL-positive cells (18.7 ± 3.1%; *n* = 5 animals) in the penumbra region, compared with stroke control group (32.8 ± 2.3%; *n* = 5 animals, *p* < .05; Figure 2(c) and (d)). Neuronal cell death was reduced by apelin-13 treatment evidenced by reduced number of TUNEL-positive cells double stained with the neuronal marker NeuN (Figure 2(c) and (e)). The protein expression of apelin receptor APLNR and the antiapoptotic gene Bcl-2 in the penumbra region of the brain was significantly higher in apelin-13-treated animals compared with those in stroke control animals (Figure 2(a), (f), and (g)). In addition, the activity of the key apoptotic enzyme caspase-3 was suppressed by apelin-13 3 days after stroke (Figure 2(a) and (h)).

### Apelin-13 Reduced Inflammation After Ischemic Stroke

**Figure 3.**
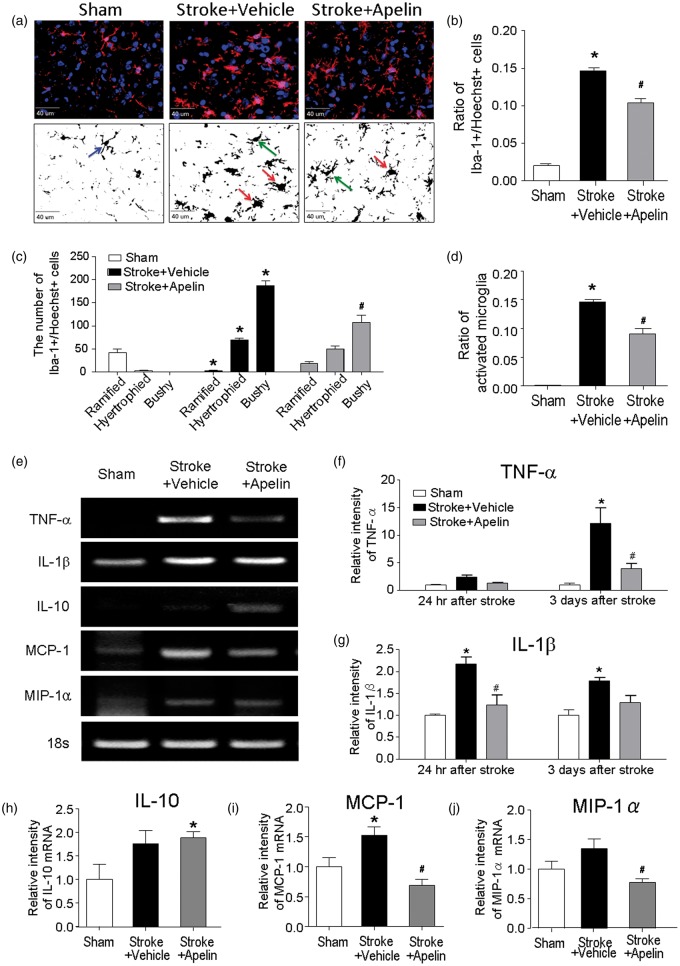
Apelin-13 attenuated inflammation in the postischemic brain. (a) Iba-1 (red) was stained to indicate the microglia recruitment and activation in the penumbra region at 3 days after stroke. Nuclei were stained using Hoechst 33342 (blue). The black and white images showed the morphology of Iba-1-positive cells generated using the threshold function of Image J software. Blue arrow indicates the representative ramified microglia, green arrow indicates the representative hypertrophied microglia, and red arrow indicates the representative bushy microglia. Pictures were taken from the penumbra region of the brain. (b to d) The ratio of Iba-1+/Hoechst+ colabeled cells in all cell population (Hoechst+ cells) (b), the number of ramified microglia, hypertrophied microglia, bushy microglia (c), and activated microglia (the total number of hypertrophied and bushy microglia) (d) were quantified in each group. All these measured cells significantly increased in stroke control animals, except that the number of ramified microglia showed in the bar graph in (c) significantly reduced compared with that in sham animals, suggesting less resting microglia after stroke. Apelin-13 treatment significantly reduced the ratio of Iba-1+/Hoechst+ colabeled cells, the number of bushy microglia, and the total number of activated microglia in the penumbra region. *p < .05 versus sham, #*p* < .05 versus stroke + vehicle; *n* = 3 in sham group, *n* = 6 in stroke + vehicle and stroke + apelin group. (e) Representative RT-PCR images of the mRNA expressions of TNF-α, IL-1β, IL-10, MIP-1α, and MCP-1 in the penumbra region at 3 days after stroke. (f to j) Quantified data showed that TNF-α showed moderate increase 24 hr after stroke (*p* < .05, one-way ANOVA but *p* > .05 two-way ANOVA) and a marked increase 3 days after stroke. Apelin-13 treatment significantly ameliorated the elevation of TNF-α mRNA level after stroke (f). IL-1β expression significantly increased and the elevation sustained until 3 days after stroke. Apelin-13 treatment significantly ameliorated this elevation (g). IL-10 expression remained about the same level after stroke; however, apelin-13 treatment significantly enhanced the level of this anti-inflammatory factor in the penumbra measured at 3 days after stroke (H). Apelin-13 treatment also showed significant reduction in the mRNA levels of MCP-1 and MIP-1α 3 days after stroke (i and j). *p < .05 versus sham, #*p* < .05 versus stroke + vehicle; *n* = 3 in sham group, *n* = 6 in stroke + vehicle group and stroke + apelin group. TNF-α = tumor necrosis factor-alpha; MIP = macrophage inflammatory protein; MCP-1 = monocyte chemoattractant protein-1; IL = interleukin.

Inflammation and microglial activation play important roles in the pathogenesis of stroke (Yenari et al., 2010). To measure the microglial activation, we evaluated the expression of ionized calcium biding adaptor molecule-1 (Iba-1) in the penumbra region at 3 days after stroke. Immunostaining results revealed that the number of Iba-1-positive cells in the stroke brain significantly increased in the penumbra region. The morphological assessment of Iba-1-positive cells revealed that in the penumbra region, there were few microglia with ramified morphology of surveillant microglia, and the majority of Iba-1-positive cells were activated microglia with hypertrophied and bushy shapes (Soltys et al., 2001). On the other hand, stroke animals received apelin-13 treatment showed significant reductions in the total number of Iba-1-positive cells as well as in the number of activated microglia (Figure 3(a) to (d)). At 3 days after stroke, there were also infiltrating macrophages recruited to the ischemic cortex, which were both F4/80 and Iba-1 positive, mostly located in the ischemic core region, with few cells in the penumbra (Supplemental Figure 1).

Because microglial activation following ischemic injury was known to release the pro- or anti-inflammatory cytokines (Lucas et al., 2006), we measured the levels of the inflammatory cytokines and chemokines in the penumbra region at 24 hr and 3 days after stroke. RT-PCR experiments showed a moderate increase in TNF-α expression 24 hr after stroke and a marked increase 3 days after stroke. Apelin-13 treatment significantly suppressed the elevation of TNF-α 3 days after stroke (Figure 3(e) and (f)). The expression of IL-1β significantly increased in the penumbra region 24 hr after stroke, and this elevated level sustained at least until 3 days after stroke. Aplein-13 treatment significantly attenuated the increase of IL-1β after stroke (Figure 3(e) and (g)). However, the expression of IL-6 was not statistically different among the sham, stroke control, and apelin-13 treatment groups (data not shown). Interestingly, the mRNA expression of the anti-inflammatory cytokine IL-10 increased in the apelin-13-treated animals compared with those in the sham group 3 days after stroke (Figure 3(e) and (h)). Besides the pro- or anti-inflammatory cytokines, we also found significantly less proinflammatory chemokines such as MIP-1α and MCP-1 in apelin-13-treated animals at 3 days after stroke (Figure 3(e), (i), and (j)). These results suggested that apelin-13 treatment could suppress microglial activation and inhibit the release of proinflammatory cytokines and chemokines after stroke. Meanwhile, it may increase the anti-inflammatory factor IL-10.

### Apelin-13 Enhanced Angiogenesis After Ischemic Stroke

**Figure 4.**
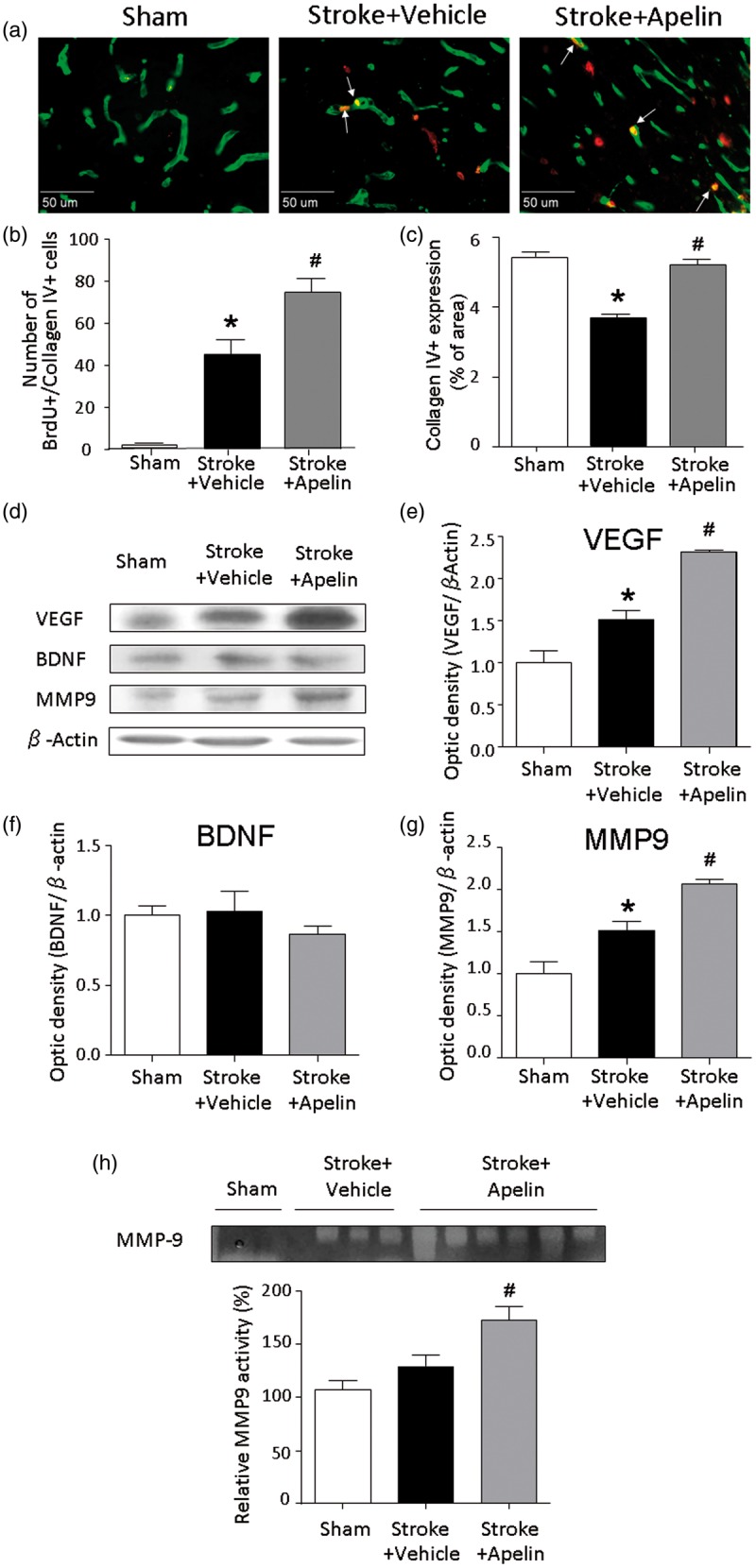
Apelin-13 enhanced the long-term angiogenesis after stroke. (a) The angiogenesis in peri-infarct region was examined using collagen IV (green) and BrdU (red) costaining at 21 days after stroke. (b) Apelin-13 treatment increased the number of collagen IV+/BrdU+ colabeled cells in the peri-infarct region of stroke animals. (c) Less collagen IV expression was found in the peri-infarct region of stroke control animals, compared with that in the sham animals, while apelin-13 treatment significantly increased collagen IV expression in the peri-infarct region 21 days after stroke. *p < .05 versus sham; #*p* < .05 versus stroke + vehicle, *n* = 3 in sham group, *n* = 6 in stroke + vehicle and stroke + apelin group. (d) Western blot assay was used to detect the protein expression of VEGF, BDNF, and MMP9 in the peri-infarct region at 14 days after stroke. β-actin was used as a loading control. (e to g) Quantified data showed that VEGF and MMP9 expression was increased by apelin-13 treatment, while the expression of BDNF was not changed. *p < .05 versus sham; #*p* < .05 versus stroke + vehicle, *n* = 3 in sham group, *n* = 4 in stroke + vehicle and stroke + apelin group. (h) Gelatin zymography was used to assess the activity of MMP9. The data showed elevated activity of MMP9 in the peri-infarct region in apelin-13-treated animals compared with those in stroke control animals 14 days after stroke. #*p* < .05 versus stroke + vehicle. *n* = 3 in sham group, *n* = 4 in stroke + vehicle, *n* = 6 in stroke + apelin group. VEGF = vascular endothelial growth factor; MMP9 = matrix metalloproteinase-9; BDNF = brain-derived neurotrophic factor.

We tested the hypothesis that apelin-13 could enhance the postischemia angiogenesis in the brain. Animals received daily injections of BrdU beginning on the Day 3 after ischemic stroke to label the newborn cells until sacrificed at 21 days after stroke. The number of BrdU-positive cells colocalized with collagen IV was examined as a marker of angiogenesis. There were significantly more BrdU+/collagen IV+ colabeled cells in the peri-infarct region in apelin-13-treated stroke animals (45.2 ± 6.7 vs. 74.5 ± 6.7 in stroke + vehicle and stroke + apelin-13 groups, respectively; *p* < .05, *n* = 6 animals each group), suggesting enhanced angiogenesis in apelin-13-treated animals (Figure 4(a) and (b)). Twenty-one days after stroke, lower collagen IV expression was found in the peri-infarct region of stroke animals compared with that in the cortex of sham animals, suggesting a deteriorating effect on the vasculature. Apelin-13 treatment resulted in significantly increased collagen IV expression in the peri-infarct region 21 days after stroke (Figure 4(a) and (c)).

To elucidate the possible mechanisms of the proangiogenic effects of apelin, we measured the expression of angiogenesis related factors in the peri-infarct region at 14 days after stroke. VEGF, BDNF, and MMP9 were measured using Western blot analysis, and the results showed that apelin-13 significantly increased the protein expression of VEGF and MMP9 in the peri-infarct region. The level of BDNF expression was similar between experimental groups (Figure 4(d) to (g)). In addition, gelatin zymography was used to assess the activity of MMP9 in the peri-infarct region. Consistent with Western blot analysis, results from this assay verified that apelin-13-treated animals showed enhanced activity of MMP9, compared with those in stroke control animals 14 days after stroke (Figure 4(h)). On the other hand, there was no significant difference in the level of MMP9 protein expression in the contralateral cortex between groups (Supplemental Figure 2(a)). To examine whether the enhanced MMP9 activity in the peri-infarct region might affect the integrity of BBB, we measured the expression of tight-junction protein occludin in this region using immunofluorescence staining at 14 days after stroke. The results showed that there was no significant difference in the expression of occludin between stroke control and apelin-13 treatment groups (Supplemental Figure 2(b) and (c)).

### Apelin-13 Improved Local Blood Flow Restoration and Functional Recovery After Ischemic Stroke

**Figure 5.**
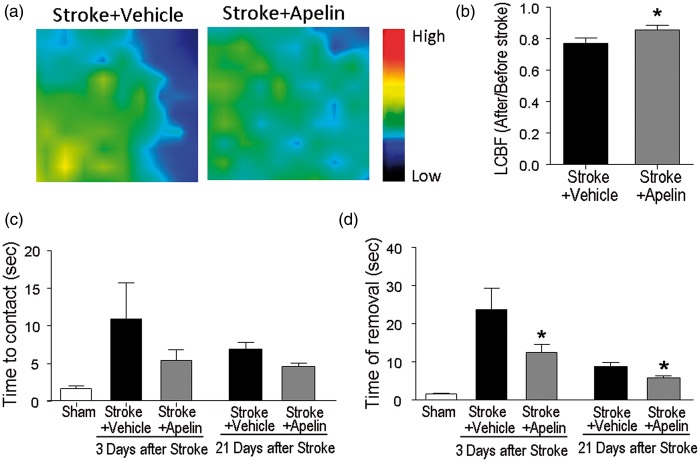
Apelin-13 promoted the long-term LCBF restoration and functional recovery after stroke. (a and b) Local cerebral blood flow (LCBF) in the penumbra region was measured at 21 days after stroke using Laser Doppler. The Laser Doppler imaging and quantified data showed that stroke animals that received apelin-13 treatment exhibited better LCBF recovery than stroke control animals. (c and d) The functional recovery was also examined at 3 and 21 days after stroke using adhesive removal test. Stroke control animals spent longer time to detect the sticky dot and take longer time to remove it. Apelin-13-treated animals tend to show shorter time in detecting the dot and performed significantly faster in removing the sticky dot compared with stroke control animals. *p < .05 versus stroke + vehicle. *n* = 5 in sham group, *n* = 12 in stroke + vehicle, *n* = 10 in stroke + apelin group.

To demonstrate that the increased angiogenesis could build functional vasculatures, we measured the LCBF using a Laser Doppler Scanner at 21 days after stroke. The scanning imaging showed that stroke animals that received apelin-13 treatment had significantly greater recovery of local blood flow compared with the stroke control animals (77.2 ± 3.5% vs. 85.9 ± 0.27% in stroke + vehicle and stroke + apelin-13 groups, respectively; *p* < .05; *n* = 10 each group; Figure 5(a) and (b)).

We next assessed if the intranasal apelin-13 delivery could improve the behavior deficits after acute stroke. To specifically examine the sensorimotor functional outcomes after stroke, adhesive removal test was performed at 3 and 21 days after stroke. After the ischemic damage to the sensorimotor cortex in the right hemisphere, animals showed significant prolonged time in response to the sticky dot attached to their left paws. Animals received apelin-13 treatment showed trends in shortening the time to contact the adhesive, and they performed significantly better in removing the dot 3 and 21 days after stroke (Figure 5(c) and (d)).

**Figure 6.**
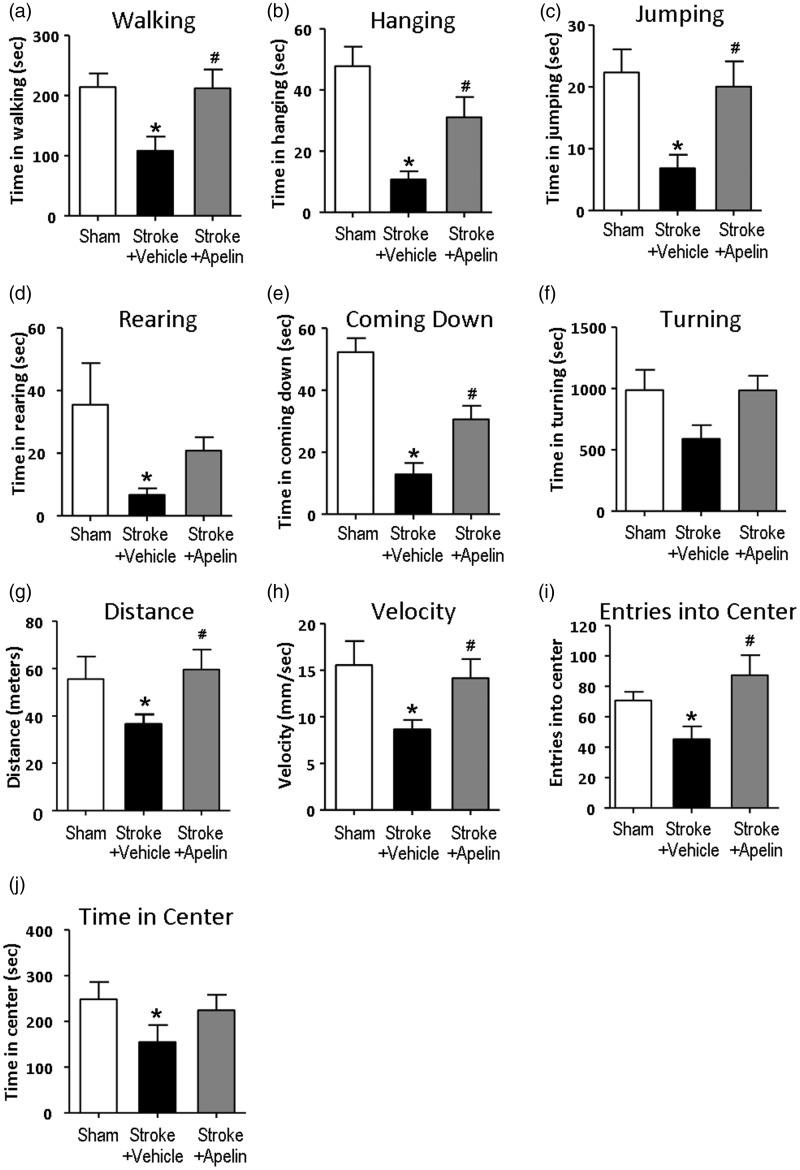
Apelin-13 improved the behavioral deficits after stroke. The behavior of animals was monitored using a HomeCageScan (a to f) and TopScan (g to j) behavioral monitoring system at 3 days after stroke. The 12-hr HomeCageScan monitoring results showed that stroke animals showed significantly less time in walking, hanging, jumping, rearing, coming down, and a trend in reduced turning behavior. And 1-hr TopScan open-field monitoring results showed a shorter travel distance, reduced velocity, fewer entries into the center area, and less time spent in the center in the stroke control group. Apelin-13-treated animals showed a similar behavior as sham animals. *p < .05 versus sham, #*p* < .05 versus stroke + vehicle; *n* = 4 in sham group, *n* = 12 in stroke + vehicle group, *n* = 10 in stroke + apelin-13 group.

A HomeCageScan monitoring system was used to measure the animal activities in their home cage environment under unconstrained and no-stress environment without human intervention. Behaviors such as walk, jump, and turn were monitored for 12 hrs for off-line analysis. Measured at 3 days after stroke, stroke control animals spent significantly less time in walking, hanging, jumping, rearing, and coming down behaviors; there was also a trend of less time in turning compared with sham animals, Apelin-13 treatment reversed the above behaviors to the level of sham animals (Figure 6(a) to (f)). Anxiety-related behaviors such as the number of entry into the center area in an open field and walking activity were recorded for 1 hr using a TopScan monitoring system. Stroke control animals showed the reduced travel distance, slower movement, fewer entries to the center area, and less time spent in the center area, suggesting a certain degree of increased anxiety after ischemia injury while the walking activity was also reduced. Intranasal delivery of apelin-13 significantly ameliorated these abnormal behaviors after stroke (Figure 6(g) to (j)).

## Discussion

The present investigation evaluated the neuroprotective effect of intranasal delivery of apelin-13 and related mechanism against ischemic stroke in a mouse model of focal cerebral ischemia, explored long-term regenerative effects on angiogenesis and functional recovery after stroke. Our results demonstrate for the first time that the neuroprotectant apelin-13 can be administered through the noninvasive intranasal delivery method, and this treatment effectively enhances the apelin level in the ischemic brain, reduces the microglia activation, attenuates inflammatory cytokines/chemokines levels, and decreases the apoptotic cell death. The results suggest that apelin-13 could be used as a neuroprotective as well as a regenerative treatment after ischemic stroke. Apelin-13 was given acutely after stroke and followed by chronic treatment of daily administration. This treatment protocol was designed based on the rationale that the noninvasive method of intranasal administration can be performed on-site to patients without much delay. The repeated chronic treatment targets the time-dependent regenerative process for lasting effect of functional recovery.

Recently, the neuroprotective effect of apelin administered through lateral cerebral ventricle injection was reported in a rat transient focal ischemia model and in a mouse neonatal hypoxia/ischemia encephalopathy model (Khaksari et al., 2012; Gu et al., 2013). In seeking for a noninvasive method to deliver apelin as a clinically feasible treatment for ischemic stroke, the intranasal route is an attractive and practical method. Drugs delivered through the intranasal route can bypass BBB and reach brain tissues by utilizing the olfactory neuronal pathways in the cribriform plate, which leads to direct nose-to-brain distribution. Previous studies have shown the efficacy of intranasal administration of proteins and peptides to the brain (Hanson and Frey, 2008; Dhuria et al., 2010; Scafidi et al., 2014). The intranasal drug delivery is highly efficient. The brain concentrations of proteins and peptides after intranasal delivery are similar or even higher comparing with systemic administration (Scafidi et al., 2014). We showed recently that intranasal delivery is also an effective method in stem cell transplantation therapy after ischemic and hemorrhagic stroke (Wei et al., 2013, 2015; Sun et al., 2015). Intranasally administered bone marrow mesenchymal stem cells were detected 1.5 hr later in the brain, and many cells migrated to the ischemic cortex (Wei et al., 2013). In the present study, 30 min after intranasal delivery of apelin-13 resulted in significant high levels of apelin in the brain detected in the ipsilateral cortex of stroke animals. These results suggest that apelin-13, as many proteins, peptides, and cells, can enter the postischemic brain via the intranasal route. Endogenous apelin is also expressed in other brain regions such as hippocampus, hypothalamus, thalamus, basal ganglia, and contralateral cortex. We did not see significant effect of apelin-13 treatment on apelin expression in these brain regions (data not shown).

To increase tissue permeability, hyaluronidase was administered to the nasal space 30 min prior to apelin-13 administration. The use of hyaluronidase has become a routine procedure in intranasal delivery of therapeutics. A possible drug–drug interaction between hyaluronidase and apelin-13 cannot be excluded. However, this possibility is unlikely because hyaluronidase was given many minutes before apelin-13. In the absence of hyaluronidase, apelin-13 has been shown to have similar neuroprotective effect *in vitro* and *in vivo* (O’Donnell et al., 2007; Zeng et al., 2010; Khaksari et al., 2012; Yang et al., 2014; Yan et al., 2015). It is also important to note that intranasal delivery can be performed in conscious animals or humans as shown in this study without the need of anesthetics. This makes the method even more clinically practical with reduced risk of side effects from repeated anesthesia.

Apoptosis have been linked to neuronal death after ischemic stroke, trauma, spinal cord injury, and some neurodegenerative diseases. It was reported that apelin-13 upregulated the expression of survival gene Bcl-2 and downregulated caspase-3 activity in cardiomyocyte apoptosis induced by glucose deprivation (Zhang et al., 2009). Our previous *in vitro* study also showed that apelin-13 blocked serum deprivation-induced cell death, mitochondria depolarization, cytochrome c release, and caspase-3 activation (Zeng et al., 2010). In the present study on stroke animals, elevated caspase-3 activation was observed in the ischemic brain at 3 days after stroke. Intranasal administration of apelin-13 significantly suppressed the caspase-3 activation and increased the survival gene Bcl-2 after stroke, providing an antiapoptotic mechanism of apelin-13 in the ischemic brain (Tang et al., 2007; Zeng et al., 2010; Yang et al., 2014).

Endangered neurons insulted by ischemia synthesize and release chemokines such as MCP-1, MIP-1α, and interferon-inducible protein, which can recruit microglia (Flugel et al., 2001; Rappert et al., 2004; Wang et al., 2008). Increased MCP-1 and MIP-1α was detected in neurons after ischemia (Che et al., 2001; Wang et al., 2008). Although the mechanisms of chemokine-mediated neuronal death are still under investigation, accumulating evidence suggests that early production of proinflammatory mediators such as TNF-α and IL-1β through the induction of chemokines contribute to ischemic cell death (Barone et al., 1997; Yamashita et al., 2000; Douglas et al., 2013). In the current study, we observed that the expressions of chemokines, such as MCP-1 and MIP-1α and proinflammatory cytokines including TNF-α and IL-1β were diminished by apelin-13 treatment. On the other hand, the antiapoptotic cytokine IL-10 was increased by apelin-13. These findings suggest that apelin-13 treatment prevents inflammation-mediated neuronal damages through regulations of inflammatory factors and activation of microglia cells after an ischemic insult.

In the present investigation, we show that apelin-13 also facilitates regenerative activities in the ischemic brain. Chronic treatment of apelin-13 increased the angiogenesis and promoted the LCBF restoration and long-term functional recovery after stroke. The improved blood flow recovery and behavioral recovery is expected to be a result of the combined benefits from neuroprotection and regeneration. Apelin-13 was given every day starting from 30 min after stroke. This experimental design targets to protect cells as well as promote persistent regeneration in the poststroke brain. Whether shorter duration of apelin-13 treatment, and the dose-response relationship or the time course of alterations of related factors need to be determined in a systemic preclinical study on the same and different stroke models.

Previous reports showed that overexpression of apelin increased Sirt3, VEGF/VEGFR2, and angiopoietin-1 (Ang-1)/Tie-2 expression and the density of capillary and arteriole density in the heart of diabetic mice (Zeng et al., 2014). On the other hand, inhibition of apelin expression switched endothelial cells from proliferative to mature state in pathological retinal angiogenesis (Kasai et al., 2013). We now demonstrate a proangiogenic role of apelin after focal ischemic stroke. The increased collagen IV expression has been shown to contribute the NO-induced angiogenesis (Wang and Su, 2011). Although we did not measure NO expression/release, the increased expression of VEGF and MMP9 in apelin-13-treated animals is in line with enhanced angiogenesis and the long-term functional recovery in apelin-13-treated animals.

In conclusion, our study shows the anti-inflammatory, antiapoptotic, and proregenerative actions of apelin-13, which can be delivered by a noninvasive, clinical feasible method of intranasal administration. For the first time, apelin-13 was shown to promote the angiogenesis and LCBF restoration after ischemic stroke, indicating the potential application of apelin-13 as a multifaceted drug for acute and chronic treatments of ischemic stroke.
